# Autopsy-detected diagnostic errors in critically ill patients with cirrhosis

**DOI:** 10.1186/cc13227

**Published:** 2014-03-17

**Authors:** N De Mey, J Wauters, A Wilmer, P Meersseman

**Affiliations:** 1 UZ Gasthuisberg, Leuven, Belgium

## Introduction

Even with the availability of new and more effective diagnostic procedures over the past decades, autopsy remains one of the most reliable methods to validate clinical diagnoses. The aim of this review was to determine whether autopsy plays a role in extending knowledge about the cause of death in patients with cirrhosis who died in the ICU.

## Methods

We conducted a retrospective review of medical records and postmortem findings in critically ill patients with cirrhosis who were admitted to a major university teaching medical ICU (MICU) between August 2007 and August 2013. Agreement between diagnoses before death and postmortem findings were compared using the Goldman system [1]. Review was independently performed by a fellow in critical care medicine and a board-certified attending. Autopsy diagnoses included histologic and microbiological findings. The records were also reviewed for demographics, APACHE II score and all performed diagnostic procedures.

## Results

Of 641 patients admitted with diagnosis of cirrhosis, 86 (13%) died in the MICU. Forty-five (52%) patients underwent an autopsy. Forty-two autopsy reports were available for review, three histologic and microbiological reports were missing. Major missed diagnoses (principal underlying disease related to death and primary cause of death itself) were present in seven patients (17%), four in class I (10%) and three in class II (7%) (Table 1). Minor missed diagnoses were present in 13 patients (31%) (class III and IV). Postmortem findings were in complete agreement (class V) with clinical cause of death in almost one-half of the patients (*n *= 19, 45%).

**Table 1:** Major missed diagnosis

Class I (*n *= 11)	Class II (*n *= 3)
1. Metastatic linitis plastica	1. Acute pancreatitis
2. Oesophageal variceal bleeding	2. Necrotizing mucor mycosis pneumonia
3. Disseminated mucor mycosis	3. Focal aspergillosis (myocardial)
4,5,6. Invasive aspergillosis (*n *= 3)	
7. Invasive candidiasis	
8. Herpes simplex pneumonia	
9. Pneumocystis pneumonia	
10. Gastric rupture	
11. Disseminated HCC, vs. porta thrombosis	

Class I = missed major diagnosis that would have changed management and might have resulted in cure or prolonged survival. Class II = missed major diagnosis that would not have modified ongoing patient care.

## Conclusion

Despite declining rates worldwide, autopsy remains an important tool for quality and safety assurance. In this retrospective study, autopsy showed that knowledge of the correct premortem diagnosis would have altered therapy in 10% of critically ill cirrhotic patients.
